# Error-based learning and lexical competition in word production: Evidence from multilingual naming

**DOI:** 10.1371/journal.pone.0213765

**Published:** 2019-03-22

**Authors:** Elin Runnqvist, Kristof Strijkers, Albert Costa

**Affiliations:** 1 Aix-Marseille Université, CNRS, Laboratoire Parole et Langage UMR, Aix-en-Provence, France; 2 Center for Brain and Cognition, UPF, Barcelona, Spain; 3 Institució Catalana de Recerca i Estudis Avançats (ICREA), Barcelona, Spain; Leiden University, NETHERLANDS

## Abstract

We tested whether learning associated to lexical selection is error-based, and whether lexical selection is competitive by assessing the after-effects of producing words on subsequent production of semantic competitors differing in degree of error (translation equivalents). Speakers named pictures or words in one language (part A), and then named the same set of pictures (old set) and a new set in another language (part B). RTs for the old set (i.e., translation equivalents) were larger than for the new set (i.e., items which not have been named previously in another language). Supporting that learning is error-based, this cost was mostly larger after naming in a language with a higher degree of error (L2 vs. L1). Supporting that lexical selection is competitive, after naming in a language with a high degree of error (L3), the cost was larger for naming in another language with a high degree of error (L2 vs. L1).

## Introduction

Speaking seldom constitutes a one to one mapping activity since most ideas can be expressed through more than one phonological form. Furthermore, closely related concepts and their corresponding words are believed to co-activate each other through spreading activation during speech production [1, 2, 3]. That is, when preparing to utter a word like *“dog”*, related representations such as *“cat”* and *“horse”* will also become (partially) activated. How are speakers able to efficiently select and produce the words that match their intentions? Models of lexical access conceive the achievement of this feat as a learning process destined to make targets or competitors more or less available respectively by strengthening the semantic to lexical connection weights of just produced targets, and/or weakening the semantic to lexical connection weights of competitors [4, 5]. For example, when uttering *“dog”*, the connections between the verbal label and its semantic features (such as ‘has eyes’) will become stronger and/or the connections from the related words *“cat”* and *“horse”* to the shared semantic features with *“dog”* (‘has eyes’) will become weaker. Still to be determined is (a) whether this learning is sensitive to the degree of error in the activation levels of targets and competitors (i.e., error-based learning). In brief, this means that there will be less lexico-semantic weight adjustments (learning) for targets with a higher resting-level of activation (and thus a lower degree of potential error such as high-frequency words) compared to targets with a lower resting-level of activation (and thus a higher degree of potential error such as low-frequency words); and (b) whether the speed and accuracy of lexical selection is affected by the activity of related words (a competitive lexical selection as a result of strengthening the lexico-semantic connections of the target word) or rather is achieved when an item reaches an absolute threshold (non-competitive selection as a result of weakening the lexico-semantic connections of semantically related non-target words). Shedding light on these issues of error-based learning and lexical competition was the aim of this study.

### Lexical competition

The presence of competition during lexical selection has led to long-standing debate. According to the lexical competition account, the more active other words (i.e., competitors) are in the system the slower and more error-prone production is (e.g., selection by competition: [3, 6–12]. An alternative view is that lexical selection simply occurs when a given word reaches a selection threshold, regardless other words’ activity level [2, 4, 13–15]. The debate has especially focused on trying to explain the semantic contextual effects observed in various experimental contexts such as the picture‐word interference (henceforth PWI) and semantic competitor priming. In the PWI paradigm, naming a picture (dog) is hampered by the concurrent presentation of a semantically related word (cat) as compared to an unrelated word (car) [16–17]. Regarding semantic competitor priming, a variety of different picture naming tasks have elicited the common observation that producing “*cat*” hampers the production of related words such as “*dog*” on a subsequent and not necessarily contiguous trial (e.g., [5, 18–24]. These patterns of semantic interference have been interpreted as revealing lexical competition. In the case of PWI, semantically related distracters are assumed to have a higher level of activity than unrelated distracters, and consequently hamper production to a larger extent. In the case of the semantic competitor paradigms, especially when considering those cases in which several trials intervene between the related items (i.e., the cumulative semantic interference paradigm), one has to assume that previous naming of an item such as “*cat*” leads to a persistent strengthening of the connection weights from semantics to lexical items, which then is summed to the more transient activation that lexical items receive through spreading activation on the trial where “*dog*” has to be named [5]. In other words, these interference effects have been accounted for by a learning mechanism where lexico-semantic strengthening of an uttered word *(“cat”)* has as consequence that this strengthened word will act as a stronger competitor when later on one wants to produce a related word *(“dog”)*. This kind of long-lasting effect has led researchers to claim that the language production process must integrate a component of learning.

However, these interpretations have been challenged. In the case of PWI, an alternative response exclusion account claims that whenever speakers face a stimulus that can afford two responses, they cannot help but preparing both, the picture name and the distractor word ([14, 25–27], but see [28]). These responses are stored in an output buffer until one of them can be excluded, and the ease of such response exclusion is sensitive to how appropriate the word is as a potential response. Thus, related distracters will be harder to exclude as a potential response than unrelated ones since they are less inappropriate responses [25–26]. Put broadly, according to this view, there is no competition during the lexical selection process, but there is competition to select the appropriate response (i.e., during decision-making after lexical processing). Turning to the semantic competitor priming, it has been argued that lexical competition might not need to be invoked to account for several interference results (e.g., cumulative semantic interference, blocked cyclic naming etc.). For example, Oppenheim et al. [4] simulated these phenomena in a model free of lexical competition that incorporates a post‐lexical learning mechanism of competitor weakening (as opposed to the strengthening mechanism explained in the previous paragraph): when producing “*cat*”, “*dog*” also becomes active but does not compete with selection of “*cat*”. After lexical selection, a learning mechanism weakens the connections between the semantic features shared by “*CAT*” and “*DOG*” and the word “*dog*”, rendering “*dog*” less accessible on a subsequent trial (words in lower case (“word”) denote lexical items, words in upper case (“WORD”) denote (lexical) concepts). Thus, according to this explanation, semantic competitor effects might be compatible with both competitive and non‐competitive selection as they could be explained either in terms of target strengthening (inducing competition) or in terms of weakening co‐activated non‐targets (and thus a non‐competitive system; do note that the computational model of Oppenheim and colleagues [4] contained both strengthening and weakening. The crucial question in the present study is which of the two mechanisms is mainly responsible for semantic competitor effects, without precluding in any way the coexistence of both). Given that the current semantic contextual effects can be accounted for regardless of whether lexical competition is embraced or not, here we seek a different way to test its presence.

### Error-based learning

The learning mechanism by which links between conceptual and lexical representations are strengthened or weakened can be described in at least the two following ways. Howard and colleagues [5] hypothesized that producing the word *“dog”* results in the strengthening between its lexical representation and the corresponding semantic features by a constant amount irrespective of the resting activation level of the lexical representation (i.e., irrespective of whether it concerns a well-known or completely novel word). Though in Howard et al. [5] no weakening is conceived, one could likewise imagine a constant weakening of all competitors regardless their potential level of interference. That is, at the same time that the lexico-semantic links of the target word *(“dog”)* are strengthened, those of potential competitors *(“cat”*, *“rat”)* would be weakened. A slightly different model proposed by Oppenheim et al. [4] assumes that the strengthening/weakening is not a constant but rather depends on the resting level activation of the target representation. In this error-based learning model, the difference between the initial activation levels of a given item and the desired activation levels (i.e., the error) of that item drives learning. For example, low frequency words (e.g., *“squirrel”*) would display a larger difference between initial activation and the threshold required for selection–this difference is referred to as error—and would thus require more strengthening when being the speaker’s target compared to high frequency words (e.g., *“dog”*). In other words, the lower the initial resting level activation of a word (such as between low vs. high frequency words), the more learning (strengthening) will occur (since there is more room for “improvement”). Similarly, when acting as competitors, low frequency words would require less weakening than high frequency words (since there is less room for “decline”). Although there is evidence for such error-based learning in other domains of cognition and even at other levels of language production [4], the error-based nature of learning has received less attention in the domain of word production.

### The current study: Testing the notions of error-based learning and lexical competition

A first goal of the present study was to test whether the learning associated to lexical selection is error-based. Concretely, we manipulated the magnitude of the error elicited by competitors and targets, and then tested the effect on subsequent naming instances. Our rationale was the following: If a given competitor has been named previously, naming an item would be difficult either because it was previously weakened or because the competitor was strengthened. Crucially, however, if learning is error-based, this difficulty in naming will increase as a function of how much the current target was weakened or its competitor was strengthened before. That is, as explained in Section 1.2, words with a low resting-level of activation (and thus high error, meaning that the possibility to make an error is high) will be strengthened more as target (there is more room for improvement) and weakened less as competitor (there is less room for connection decrease) compared to words with a higher resting-level of activation (and thus low error, meaning that the possibility to make an error is lower). Let us try to exemplify this with translation words (the type of stimuli we will use in the current experiments): Words in a bilingual’s dominant language (first language, L1) are generally stronger (higher resting-level of activation) than the words in their non-dominant language (second language, L2); as evidenced by the slower reaction times (RTs) for L2 picture naming than L1 picture naming [29–32]. In the context of the current study this means that an L1 word (e.g., *“perro”*; *“dog”* in Spanish) has less error (as target) than its translation equivalent in L2 (e.g., *“gos”* in Catalan). If the lexico-semantic connections between a word and its concept are subject to error-based learning, naming the L1 word *“perro”* will cause less strengthening between its semantic features and the lexical representation (because resting-level activity is already high) and/or less weakening between the shared semantic features and the lexical representation of its translation *“gos”* (because the resting-level activity of *“gos”* is already lower than of *“perro”*) compared to when naming the L2 word *“gos”* (which will be strengthened more because it has higher error and/or weaken the translation more because the L1 word has lower error). As a consequence of such error-based learning dynamic, the impact of naming a word in L1 upon subsequent naming of its translation in L2 will be different compared to the impact of naming a word in L2 upon subsequent L1 naming. Concretely, L2 naming *(“gos”)* will cause more interference for subsequent L1 naming *(“perro”)* than vice versa. In contrast, if weight adjustments between semantic features and the lexical item to which they are connected do not consider the degree of error, one predicts that the impact of naming in L1 upon subsequent L2 naming will be the same as naming in L2 upon subsequent L1 naming. This is exactly what we set out to test in experiments 1a and 1b. Advancing upon the results, we obtained evidence in experiments 1a and 1b for error-based learning (that is, more interference for the naming direction L2 -> L1 than L1 -> L2).

Hence, in a second part of the study, we aimed to test whether the error-based learning effects observed in experiments 1a and 1b are (mainly) driven by strengthening, and thus require a competitive framework to explain the interference effects, or by weakening, which can explain interference effects without the presence of lexical competition. Concretely, our rationale was that if naming an item is difficult because of previous competitor strengthening, such difficulty should be larger when the competitor had a high error as target and thus underwent more strengthening (i.e., low-frequency competitors). If naming an item is difficult because it has been weakened previously when naming a related object, such difficulty should be greater for items that had a low error as competitors and thus underwent more weakening (i.e., high frequency competitors). To continue with the above example: If a word is named in a weak L3 *(“dog”)*, in terms of strengthening, the lexico-semantic weight changes will be substantial rendering it a strong competitor for subsequent naming in L2 or L1. Given that the L2 translation *(“gos”)* is already a weaker representation than the L1 translation *(“perro”)*, it logically follows that L2 naming will suffer more from the previous L3 strengthening than L1 naming. However, and interestingly, if weakening is the main responsible behind interference effects, the prediction here is the reverse: After naming the L3 word *(“dog”)*, the lexico-semantic connections between the L1 translation *(“perro”)* and its shared concept *(“DOG”)* will be weakened more compared to the L2 translation *(“gos”)* and the shared concept *(“DOG”)*. This is because during L3 naming, the L1 translation is a stronger competitor than the L2 translation (given that words in L1 have higher resting-level of activation), consequently producing more lexico-semantic weight changes (weakening) than for the already weaker L2 word. In this case, the prediction is that after L3 naming there will be larger translation interference effects for L1 than L2. This is exactly what we will test in Experiment 2. Note also that making use of such trilingual design offers the opportunity to pitch the predictions of weakening against those of strengthening. This is important, given that we know of no current study on semantic interference in language production that allows doing so (that is, in all those cases strengthening and weakening lead to the same predictions).

**Fig 1:**
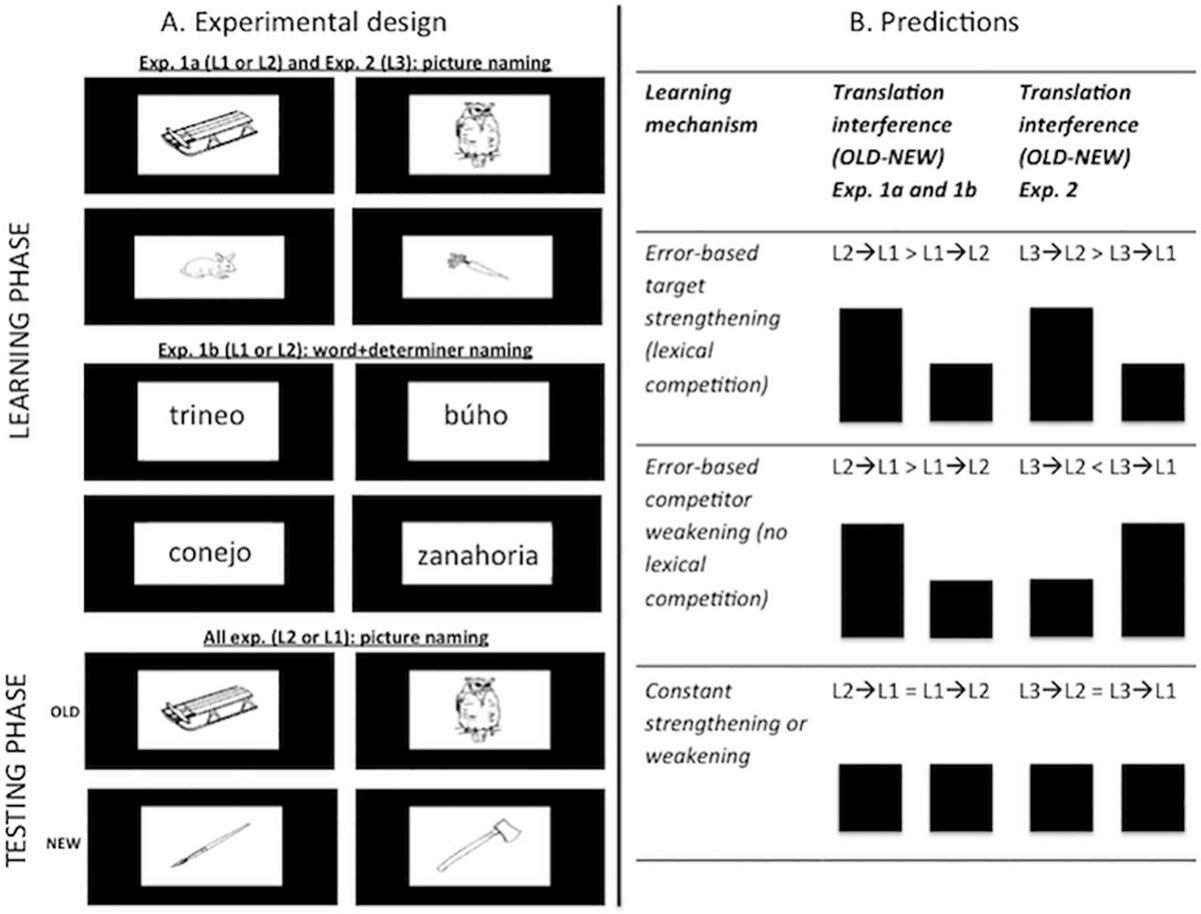
Schematic representation of the experimental design (left) and the predictions of experiment 1a, 1b and 2 in what regards the relative magnitude of translation interference effects for non-cognates.

To sum up the currents study’s rationale and approach: For both parts of the study, we tested our predictions using as targets and competitors translation words, which are strong competitors given their semantic overlap (note furthermore that we are testing early highly-proficient Spanish-Catalan bilinguals, for which it is safe to say that translation words of the type used here–concrete nouns–have identical concepts). Importantly, due to differences in language proficiency and/or frequency of use, translation words also differ in their degree of error: As targets, L1 words have a smaller degree of error (i.e., highest resting levels of activation) than L2 (i.e., medium resting levels of activation) or L3 words (i.e., lowest resting levels of activation). The latter provides suitable conditions to test our predictions by assessing how difficult it is to name a word in language whose translation has been named before in another language (see Fig 1) [33–35]. By using these advantageous properties inherent to bilingualism (i.e., same semantics linked to lexical representations with different degree of error), in experiments 1a and 1b we will explore whether error-based learning dynamics are indeed functional in language production by assessing whether naming in L2 and subsequently in L1 produces stronger translation interference than naming in L1 and subsequently in L2. Please note that at this point we remain silent whether the driving force behind such potential differential interference is weakening (non-competitive) or strengthening (competitive). The latter will be assessed in experiment 2 by making use of a trilingual design where a (competitive) strengthening account predicts more translation interference from L3 naming on L2 naming, while a (non-competitive) weakening account predicts more translation interference from L3 naming on L1 naming.

## Experiments 1a and 1b: Testing the error-based nature of learning

In these experiments Spanish‐Catalan bilinguals first (Learning phase) named pictures (Experiment 1a) or words (Experiment 1b) either in L1 or in L2, and then (Testing phase) named the same set of pictures (old set) and a new set (i.e., pictures which have not been named in the Learning phase) in their other language. Contrasting RTs of the old and new sets provided us with a measure of the naming difficulty referred to in the predictions, namely *translation interference*. The bilingual design had the advantage of involving semantically identical representations with different lexical realizations (one in each language) that differ in their degree of error (L1 words have lower error than L2 words). Note that with ‘degree of error’ of a word, we refer to its error as naming target (that is, uttering an L1 word as target has lower error than uttering an L2 word as target). Due to the higher error in L2 production compared to L1 production in the Learning phase, an error-based account of learning [4] predicts larger translation interference effects in L1 compared to L2 in the Testing phase. In contrast, if degree of error plays no role (learning is a constant; [5]), the translation interference effects are predicted to be the same in L1 and L2. The purpose of including Experiment 1b with word naming in the Learning phase (instead of picture naming as in experiment 1a) was to have a measure of the impact of repeating the same visual input across the Learning and Testing phases in Experiment 1a. This is important, since previous studies have observed that when speakers name pictures that they have previously named in another language, response times are shorter (i.e., translation priming) and not longer as predicted if there was translation interference ([33, 35–36], but see [37]). We hypothesize that in these studies that included a single presentation of the pictures in each language, no translation interference could be observed because of the visual repetition priming of the old items. However, in a design including several repetitions, this should not be the case for subsequent repetitions where also the “new” items would be repeated. Hence, we included three repetitions and predicted (a) an experiment by interference interaction in the first presentation (i.e., facilitation in Experiment 1a and interference in Experiment 1b), and (b) the above-mentioned language by interference interactions for the second and third repetitions (i.e., more translation interference for L1 than for L2 naming).

### Methods

The ethics committee of clinical research Parc Salut Mar approved this research (n° 201 1/4440/I).

#### Participants

59 undergraduate students of the University of Barcelona took part in Experiment 1a and 60 undergraduate students of the University of Barcelona took part in Experiment 1b. All participants of this and the following experiments were Spanish‐Catalan bilinguals for whom Spanish was the first and dominant language according to self-report through a language history and proficiency questionnaire completed at the beginning of the experimental session ([38] see S1 File). Oral consent was obtained from all participants before their participation in the study.

#### Materials, design and procedure

In Experiment 1a, speakers were instructed to name black and white line drawings as rapidly and accurately as possible using a single word. The word-stimuli were of two different types, namely non-cognates (words without shared phonology across translation equivalents; e.g., *perro–gos*; dog) and cognates (words with shared phonology across translation equivalents; e.g., *gato–gat*; cat). The reasons to include cognates were threefold: First, the stimuli used in this experiment were (mainly) drawn from the study by Strijkers et al. [30], since this is a well-balanced stimuli-set which reliably produces a language effect (that is, faster naming latencies in L1 than L2; an important prior for the current study). In that stimuli-set cognate-status of the words was balanced (half cognates, half non-cognates). Second, including 50% of cognates has the advantage that it mimics closely the degree of similarity across languages for Spanish-Catalan bilinguals, making it a representative stimuli-set for the bilingual population of the current study. Finally, pitching cognates and non-cognates against each other could be theoretically interesting, since results may very well be different depending on the degree of form overlap. That is, the predictions we generated above hold for non-cognates (namely the same semantics linked to ‘different’ lexical realizations), but not necessarily for cognates (the possibility that the same semantics are linked to ‘overlapping’ lexical realizations; see [39]).

In this manner, 96 pictures were divided into three groups of 16 cognate names and 16 non-cognate names pair-wise controlled for lexical frequency (see S1 Table). Six experimental lists were created by (a) making all possible combinations with two (out of the three) stimuli groups (1‐2, 2‐3, 3‐1); and (b) making all possible sequences of those combinations (1‐2 followed by 2‐3 or 3‐1; 2‐3 followed by 1‐2 or 3‐1; and 3‐1 followed by 1‐2 or 2‐3). The experiment consisted of two parts (e.g., combination 1‐2 and combination 2‐3) henceforth referred to as the Learning phase and the Testing phase. Participants named the pictures of the Learning phase in one language (e.g., combination 1‐2 in L1) and those of the Testing phase in the other language (e.g., combination 2‐3 in L2). Note that, by administering the pairs of experimental lists across the Learning and Testing phases as such, in the Testing phase there is always one list that corresponds to items previously named in the other language (‘old items’; in the example here, list 2) and one list of items which have not been previously named (‘new items’; in the example here, list 3). This allows us to contrast the RTs of the ‘old items’ versus those of the ‘new items’ in order to calculate the translation interference effect (see also below). This also explains why we made three groups of stimuli (containing 32 words each) and combined them pairwise in 6 experimental lists (namely to always have one overlapping and one non-overlapping list of items between Learning and Test phase).

30 participants named in L1 first and 29 in L2 first. Within the Learning and the Testing phases, pictures were repeated three times in separate blocks and in a random order. The complete experiment consisted of 384 trials (64*3 in the Learning phase and 64*3 in the Testing phase). The experiment was administered on computers running DMDX [40]. Each trial consisted of a blank screen (700 ms), a fixation cross (700 ms), another blank screen (500 ms) and a picture (3000 ms or until response detection). Response times were recorded by DMDX’s voice key.

To assess the impact of repeating the pictures across the experimental parts, in Experiment 1b participants read aloud written words in the Learning Phase, and then named pictures in the Testing phase. Thus, in the Learning phase, speakers saw written words and were instructed to name them using their gender-marked indefinite article to ensure lexical access [15, 22, 41]. Words were presented in white font (arial size 10) on a black background. Words were preceded by a fixation cross for 1000 ms and remained on the screen for 2000 ms or until detection of a response. Everything else was equal to Experiment 1a.

The design of experiments 1a and 1b allowed us to examine the effects of experiment (1a vs. 1b), language (L1 vs. L2), cognate status (cognates vs. non-cognates) and repetition (1, 2 and 3). Of particular interest for our predictions was to examine the interactions between language and priming as well as experiment and priming in each of the three repetitions, in particular for non-cognates.

#### Analyses

Response times and error-rates of the Learning phase were included in separate repeated-measures ANOVAs with Repetition (1, 2, 3) and Cognate status (cognates vs. non-cognates) as within subjects variables and Experiment (1a vs. 1b) and Language (L1 vs. L2) as between subjects variables. For the Testing phase, the additional within subject variable Priming (old vs. new items) was included. Crucially, for the Testing phase, planned comparisons were carried out examining cognates and non-cognates separately for each repetition in order to focus on the effects and interactions of interest for our predictions (i.e., priming by language, priming by experiment). Five items (axe, bucket, door-knob, lettuce, peach) were removed from the analyses of the Testing phase due to empty cells. Because our predictions only concerned naming speed, detailed results and analyses of error-rates are reported in the Supplementary Information (see S2 File).

### Results

#### Learning phase

Mean response times for each condition are reported in Table 1. Correct trials corresponded to 95% of the observations (91% for picture naming; 98,5% for word naming). Pictures were named faster in L1 than in L2, especially in the first repetition. Words also showed a small difference between L1 and L2 naming that was larger for the first repetition. Cognates were named faster than non-cognates in both experiments, though the effect was larger for pictures than for words.

**Table 1:** RTs for the Learning phase in Experiment 1a (P)ictures) and b (W)ords). Numbers in parenthesis represent the standard error.

	L1 1^st^ rep.	L1 2^nd^ rep.	L1 3^rd^ rep.	L2 1^st^ rep.	L2 2^nd^ rep.	L2 3^rd^ rep.
P non-cog	910 (24)	772 (21)	727 (16)	1025 (23)	824 (20)	764 (15)
P cog	863 (23)	727 (18)	692 (16)	976 (22)	772 (17)	714 (15)
W non-cog	547 (23)	500 (20)	487 (15)	572 (23)	519 (20)	495 (15)
W cog	533 (22)	500 (17)	486 (15)	555 (22)	509 (17)	494 (15)

In the statistical analysis there were significant interactions between Repetition, Experiment and Language (F1(2, 228) = 4.294, MSE = 6427.075, p = .026); F2(2,752) = 13.791, MSE = 7616.520, p < .001), and between Cognate status and Experiment (F1(1,114) = 25.113, MSE = 2706.248, p < .001; F2(1,376) = 6.811, MSE = 30178.043, p = .009). To examine these interactions the experiments were analyzed separately, revealing that the Repetition by Language interaction was significant by subjects and items for picture naming (F1(2,112) = 7.475, MSE = 11025.642, p = .003; F2(2,376) = 10.285, MSE = 14483.608, p = .001) but only by items for word naming (F1(2,116) = 1.032, MSE = 2184.618, p = .347; F2(2,376) = 5.422, MSE = 841.358, p = .007). One-way ANOVAs for each repetition in the picture naming experiment revealed that the language effect was most robust in the first repetition (F1(1,56) = 9.399,p = .003; F2(1,190) = 12.406, p = .001) while only significant by items in the second (F1(1,56) = 2.580,p = .114; F2(1,190) = 6.346, p = .013) and third (F1(1,56) = 1.608,p = .210; F2(1,190) = 4.489, p = .035) repetitions. The same analysis for the word naming experiment revealed that over repetitions, the language effect was only significant by items (rep1 F1(1,58) = 1.101, p = .298; F2(1,190) = 16.460, p < .001; rep2 F1(1,58) = .465, p = .498; F2(1,190) = 13.162, p < .001; rep3 F1(1,58) = .191, p = .664; F2(1,190) = 6.160, p = .014). The cognate effect was significant in both experiments (picture naming: F1(1,56) = 36.421, MSE = 5146.982, p < .001;F2(1,188) = 9.068, MSE = 58777.975, p = .003; word naming: F1(1,58) = 13.486, MSE = 349.677, p = .001; F2(1,88) = 5.013, MSE = 1578.111, p = .026).

#### Testing phase

Mean response times and priming effects are reported in Tables 2 and 3. Correct trials corresponded to 91% of the observations (Experiment 1a 92,7%; Experiment 1b 89,6%). Old non-cognate items were named slower than new non-cognate items, especially for L1 naming. In Experiment 1a (picture naming in both phases), this pattern was only apparent from the second repetition onwards, while in Experiment 1b (word naming in Learning phase and picture naming in Testing phase) the pattern was similar across all three repetitions. In contrast, old cognate items were named faster than new cognate items, especially for the group naming in L2.

**Table 2:** RTs and translation interference (TI, negative numbers) or priming (TP, positive numbers) for the Testing phase in Experiment 1a (picture naming in both phases). Numbers in parenthesis represent the standard error.

**Non-cog**	**L1 1**^**st**^ **rep.**	**L1 2**^**nd**^ **rep.**	**L1 3**^**rd**^ **rep.**	**L2 1**^**st**^ **rep.**	**L2 2**^**nd**^ **rep.**	**L2 3**^**rd**^ **rep.**
NEW items	960 (33)	759 (21)	716 (22)	1051 (36)	848 (24)	789 (21)
OLD items	938 (32)	795 (25)	783 (21)	952 (30)	853 (25)	814 (23)
TI/TP	22 (29)	-35 (17)	-67 (14)	99 (39)	-5 (17)	-25 (16)
**Cog**	**L1 1**^**st**^ **rep.**	**L1 2**^**nd**^ **rep.**	**L1 3**^**rd**^ **rep.**	**L2 1**^**st**^ **rep.**	**L2 2**^**nd**^ **rep.**	**L2 3**^**rd**^ **rep.**
NEW items	889 (24)	711 (22)	678 (18)	1026 (34)	813 (23)	768 (19)
OLD items	739 (24)	686 (20)	668 (21)	874 (27)	756 (24)	744 (24)
TI/TP	149 (20)	26 (13)	10 (11)	151 (26)	56 (19)	24 (20)

**Table 3:** RTs and translation interference (TI, negative numbers) or priming (TP, positive numbers) for the Testing phase in Experiment 1b (word naming in Learning phase and picture naming in Testing phase). Numbers in parenthesis represent the standard error.

**Non-cog**	**L1 1**^**st**^ **rep.**	**L1 2**^**nd**^ **rep.**	**L1 3**^**rd**^ **rep.**	**L2 1**^**st**^ **rep.**	**L2 2**^**nd**^ **rep.**	**L2 3**^**rd**^ **rep.**
NEW items	839 (22)	725 (21)	701 (19)	980 (21)	827 (27)	757 (18)
OLD items	874 (29)	754 (25)	732 (20)	1033 (29)	816 (18)	775 (17)
TI/TP	-35 (24)	-29 (17)	-31 (11)	-53 (31)	11 (24)	-18 (13)
**Cog**	**L1 1**^**st**^ **rep.**	**L1 2**^**nd**^ **rep.**	**L1 3**^**rd**^ **rep.**	**L2 1**^**st**^ **rep.**	**L2 2**^**nd**^ **rep.**	**L2 3**^**rd**^ **rep.**
NEW items	774 (21)	705 (20)	667 (20)	974 (28)	784 (17)	752 (17)
OLD items	789 (24)	694 (20)	645 (16)	937 (28)	755 (19)	718 (15)
TI/TP	-15 (17)	11 (19)	21 (12)	37 (25)	29 (19)	34 (17)

In the first global statistical analysis all five variables interacted, although only significantly so in the analysis by subjects (F1(2, 230) = 3.474, MSE = 4304.335, p = .036; F2(2, 712) = 2.328, MSE = 11821.643, p = .108). As a next step, we examined cognates and non-cognates separately for each repetition in order to focus on the effects and interactions of interest for our predictions (that is, for the non-cognates in particular; see above).

For non-cognates, in the first repetition there was a significant interaction between experiment and priming (F1(1,115) = 11.495, MSE = 14143.137, p = .001; F2(1,172) = 14.361, MSE = 29736.914, p < .001), a significant main effect of language (F1(1,115) = 16.382, MSE = 37476.322, p<001; F2(1,172) = 9.966, MSE = 127153.134, p = .002), a marginally significant main effect of experiment by subjects only (F1(1,115) = 3.010, MSE = 37476.322, p = .085; F2<1) and marginally significant interactions between experiment and language also by subjects only (F1(1,115) = 3.761, MSE = 37476.322, p = .055; F2(1,172) = 1.404, MSE = 127153.134, p = .238). In the second repetition, there was a marginally significant interaction between language and priming by subjects only (F1(1,115) = 3.532, MSE = 5252.409, p = .063; F2(1,172) = 2.224, MSE = 10713.305, p = .138). In the third repetition, there was a significant interaction between language and priming by subjects only (F1(1,115) = 4.348, MSE = 2655.532, p = .039; F2(1,172) = 2.085, MSE = 6772.890, p = .151).

For cognates, in the first repetition there was a significant interaction between experiment and priming (F1(1,115) = 39.034, MSE = 7367.165, p < .000; F2(1,184) = 23.242, MSE = 20126.078, p<001) and a significant main effect of language (F1(1,115) = 41.643, MSE = 34307.736, p < .001; F2(1,184) = 31.170, MSE = 70854.740, p<001). In the second repetition, there was a main effect of priming (F1(1,115) = 11.684, MSE = 4740.206, p = .001; F2(1,184) = 14.368, MSE = 6801.090, p<001), and a main effect of language (F1(1,115) = 17.490, MSE = 20836.130, p < .000; F2(1,184) = 28.087, MSE = 20160.942, p<001). In the third repetition, there was a main effect of priming (F1(1,115) = 8.491, MSE = 3455.268, p = .004; F2(1,184) = 5.762, MSE = 6564.059, p = .017), and a main effect of language (F1(1,115) = 21.817, MSE = 17932.559, p < .000; F2(1,184) = 46.685, MSE = 13381.029, p<001).

### Discussion

The goal of Experiments 1a and 1b was to test whether learning associated to lexical selection is error-based. To this end, participants named pictures or words that either had high or low error as targets (and whose translations consequently had either low or high error), and then produced the translation equivalents in a separate naming block.

Naming translation equivalents without cross-language phonological overlap (non-cognates) was more difficult in L1 after having named in L2 than vice versa (from the second repetition onwards in experiment 1a and from the first repetition onwards in experiment 1b); a modulation that is only consistent with the notion of error-based learning. That is, the amount of target-strengthening or “competitor”-weakening is dependent upon the degree of error (linked to resting-level activation) of the target and/or semantic “competitor”. Thus, this finding allows us to integrate error-based learning as an a priori assumption in our next experiment and focus on the competitive nature of lexical selection (that is, is the mechanism responsible for the observed translation interference mainly driven by target strengthening in a competitive lexical system or “competitor” weakening in a non-competitive lexical system).

Prior to entering into the details of Experiment 2, a few words on the impact of repeating the visual input across the experimental parts (i.e., from the Learning phase onto the Testing phase). To assess this potential impact, in Experiment 1b we changed the input modality in the first part to written words, while the second part remained with picture input. While in Experiment 1a we only observed translation interference in repetitions two and three (as indicated by the priming by repetition interaction), no such modulation was present in Experiment 1b (we observed translation interference for all three repetitions). Presumably in Experiment 1a, the repetition of visual input transiently facilitated certain aspects of processing (e.g., picture recognition, conceptual identification), masking any inhibitory effects in the first presentation of part B. Note that previous studies using a similar blocked naming paradigm also observed facilitatory effects [33, 35]. However, because they only included one repetition they were unable to tease apart the contributions of visual facilitation and translation interference. Our results support a transient nature of the facilitatory effect of picture repetition and a long-lasting effect of translation interference (see also [37]), just as expected under a learning account of the latter effect. One may question whether in word plus determiner naming semantic mediation is necessary (and thus whether the paradigm is sensitive to lexico-semantic weight changes [15]). The data of experiment 1b clearly oppose such objection and instead agree with the vast amount of psycho- and neurolinguistic literature demonstrating that written (real) words (whether produced or read) do access the semantic system (even if semantics is not necessary for the task) [22, 42–47]. This does not necessarily mean lexical access and selection between object naming and word plus determiner naming is identical (for example, we do observe that the translation interference is larger for object than word plus determiner naming), but at the least word plus determiner naming does activate the semantic system in the presence of lexical activation and this is a sufficient condition to cause lexico-semantic weight changes (learning). Hence, because of the presence of semantic activation in the absence of input repetition in Experiment 1b, we observe translation interference across the board, while in Experiment 1a we only observe it from the second repetition onwards when repetition priming from the repeated picture-input between the learning and testing phases has dissipated (and thus only translation interference remains).

Finally, in both experiments we observed that while producing non‐cognates hampered subsequent translation production (especially in L1), producing cognates facilitated subsequent naming of translations. This reduced translation interference for cognates compared to non-cognates might be present simply due to the default RT advantage for cognates compared to non-cognates [30, 48–50]. That said, and despite the expected “counter-effect” that cognate facilitation would have on the potential translation interference, one may still wonder why we nonetheless did not observe a priming by language interaction (as was the case for the non-cognates). That is, even though the net effect of cognate facilitation may outweigh that of translation interference on the RTs (resulting thus overall in a facilitation effect), it still would have been possible to observe an interaction with language in that the cognate facilitation effect would be smaller in the L2->L1 direction than vice versa (and thus qualitatively display a similar result as for the non-cognates). While this would be indeed one possible prediction for cognates, it is not the only prediction. It depends, namely, on how cognates are organized and represented in the bilingual lexicon. For example, one account lending from the notion that words are Hebbian cell-assemblies in the brain [51–52], argues that cognate representations are reflected in the brain as the binding of overlapping semantic features with overlapping phonological features (and where the degree of overlap depends on the degree of formal overlap, meaning that identical cognates have a single word representation for both languages of a bilingual) [39]. In such framework, one does not expect (or only minimal) translation interference for cognates given that for the most part the same word connections (i.e., semantic-to-phonologic feature connectivity) between L1 and L2 are strengthened/weakened (note that a similar logic can in fact apply to interactive activation models where there is long-lasting feedback from phonological processing to lexical selection–i.e., the phonemes that overlap across translations would feedback activation to lexical entries in both languages. The more overlap as in the case of cognates, the more feedback and thus dissipating differences at the lexical level between L1 and L2 for this class of words: [2, 30, 53–54]. That said, with the current data we cannot go beyond speculation about how cognates are organized in the bilingual brain (nor is it the main purpose of this study). Therefore, beyond pointing out that the differential findings for cognates compared to non-cognates need not be surprising and may perfectly fit within the same error-based account (as mentioned above), we will not speculate any further about the causes of this effect and only use non-cognate stimuli for Experiment 2.

## Experiment 2: Investigating the competitive nature of lexical selection

Having established that learning is error-based, our next experiment aimed at testing whether lexical selection is a competitive process or not. That is, we aimed at distinguishing between an error-based strengthening of targets as opposed to an error-based weakening of competitors as the mechanism behind the translation interference observed in experiments 1a and b. To this end, participants named pictures in their third language (L3 English) in the Learning phase, while in the Testing phase they named the same set of pictures (old set) or a new set either in their first (group A; L1 Spanish) or their second language (group B; L2 Catalan). The trilingual design had the advantage of involving semantically identical representations with different lexical realizations (one in each language) that differ in degree of error (recall: strong representations have low error and weak representations have high error): L1 words have the lowest degree of error, L2 words a little higher, and L3 words the highest. This design provides suitable circumstances to contrast the predictions derived from strengthening versus weakening being responsible for the observed translation interference, and ultimately to test whether or not lexical selection is by competition since only weakening (as the main mechanism behind the observed translation interference) is consistent with a non-competitive account.

More concretely, if target strengthening taking place during the Learning phase leads to an increased lexical competition when naming translation words in the Testing phase, then the impact of such competition should be larger when naming in the higher error L2 compared to the inherently low error L1. Put differently, if after naming a word in L3 that word becomes a stronger competitor (due to the strengthening), then the competing activity from that L3 word will slow down the lexical selection of its L2 translation more than its L1 translation, given that L2 words are weaker memory representations to begin with than L1 words (analogous to the fact that weaker representations are more easily affected by brain damage or attrition than strong representations; [55–56]). As a result, translation interference should be larger for participants naming in L2 compared to those naming in L1 (for example, lateral inhibition would be a straightforward manner to computationally implement such effect [5, 24]). In contrast, if there is weakening of competitors, the prediction is the opposite: during L3 speech, L1 words are stronger competitors and consequently more strongly weakened than L2 words. Consequently, translation interference should be larger for participants naming in L1 compared to those naming in L2.

### Methods

The ethics committee of clinical research Parc Salut Mar approved this research (n° 201 1/4440/I).

#### Participants

54 undergraduate students of the University of Barcelona took part in Experiment 1 (27 in each group). Oral consent was obtained from all participants before their participation in the study.

#### Materials, design and procedure

Aside from the exceptions specified in what follows, everything was equal to Experiment 1a. The target words were 48 non-cognate picture names (see S1 Table). Before the experiment, participants were instructed to name all the pictures of the Learning phase in English. After this familiarization phase the experimenter provided them with the correct names if necessary. In the Learning phase of the experiment, all speakers named pictures in their L3 (English). In the Testing phase, half of the speakers named the pictures in L1 and the other half in L2. The complete experiment consisted of 192 trials (32*3 in the first part and 32*3 in the second part).

Within the Testing phase, this design allowed us to examine the effects of language (L1 vs. L2), repetition (1, 2 and 3) and, crucially, we were able to compare the items that had been named also in the Learning phase (i.e., old items) with those that had not (i.e., new items), allowing us to assess translation interference effects and their interactions with the variables of language and repetition.

#### Analyses

Response times and error‐rates were included in separate repeated‐measures ANOVAs with repetition (1, 2, 3) as within subjects variable. For the analyses of the Testing phase, the within subject variable priming (old vs. new items) was also included. The learning phase included the between subject variable group (L3→L1 vs. L3→L2) and the Testing phase included the between subject variable language (L1 vs. L2). Two items (“*fox*” and “*kite*”) were removed from the analysis of the Testing phase because of empty cells. Detailed results and analyses of error-rates are reported in the Supplementary Information (see S2 File).

### Results

#### Learning phase

Mean response times for each condition are reported in Table 4. Correct trials corresponded to 89% of the observations. Pictures were named faster with each repetition. This was supported by a main effect of Repetition in the statistical analysis (F1(2,104) = 113.997, MSE = 5534.609, p < .001; F2(2,188) = 189.088, MSE = 5088.886, p < .001). No other effects or interactions were significant (all Fs < .887, all ps>.351).

**Table 4:** RTs for the Learning phase in Experiment 2. Numbers in parentheses represent the mean standard error.

L3 1^st^ rep.	L3 2^nd^ rep.	L3 3^rd^ rep.
948 (23)	813 (18)	767 (16)

#### Testing phase

Mean response times and priming effects for each condition are reported in Table 5. Correct trials corresponded to 93% of the observations. From the second repetition onwards, participants were slower naming old items than new items. Regardless of repetition, this translation interference effect was larger for the participants naming in L2 than for those naming in L1.

**Table 5:** RTs and translation interference (TI, negative numbers) or priming (TP, positive numbers) for the Testing phase in Experiment 2. Numbers in parentheses represent the mean standard error.

	L1 1^st^ rep.	L1 2^nd^ rep.	L1 3^rd^ rep.	L2 1^st^ rep.	L2 2^nd^ rep.	L2 3^rd^ rep.
NEW items	803 (18)	648 (13)	630 (12)	987 (33)	725 (19)	688 (16)
OLD items	767 (24)	687 (16)	677 (16)	1025 (34)	847 (31)	787 (25)
TI/TP	36 (17)	-39 (16)	-47 (15)	-38 (27)	-123 (27)	-99 (21)

This pattern was confirmed in the statistical analyses by an interaction between Priming and Language (F1(1, 52) = 8.879, MSE = 11123.613, p = .004; F2 (1, 90) = 10.197, MSE = 16833.346, p = .002). When further examined, it was revealed that priming was only significant for the group naming in L2 (L1: F1(1, 26) = 1.847, MSE = 6218.608, p = .186; F2(1, 45) = 1.280, MSE = 11220.639, p = .264; L2: F1(1, 26) = 18.984, MSE = 16028.617, p < .001; F2(1, 45) = 22.191, MSE = 22446.054, p < .001). Priming also interacted with Repetition (F1(2, 104) = 14.100, MSE = 2253.596, p < .001; F2 (2, 180) = 14.410, MSE = 9424.514, p < .001), such that it was only significant in repetitions two and three (R1:F1<1; F2<1); R2: F1(1, 52) = 23.957, MSE = 7405.151, p < .001; F2(1, 90) = 46.039, MSE = 6841.668, p < .001; R3: F1(1, 52) = 30.305, MSE = 4787.322, p < .001; F2(1, 90) = 36.540, MSE = 6674.113, p < .001). There was also a significant interaction between Repetition and Language (F1(2, 104) = 24.108, MSE = 8233.812, p < .001; F2(2, 180) = 21.488, MSE = 16271.552, p < .001), indicating that the language effect (faster RTs in L1 than in L2) got smaller over repetitions. The remaining interaction between Priming, Repetition and Language was not significant (F = .507, p = .576).

### Discussion

**Fig 2:**
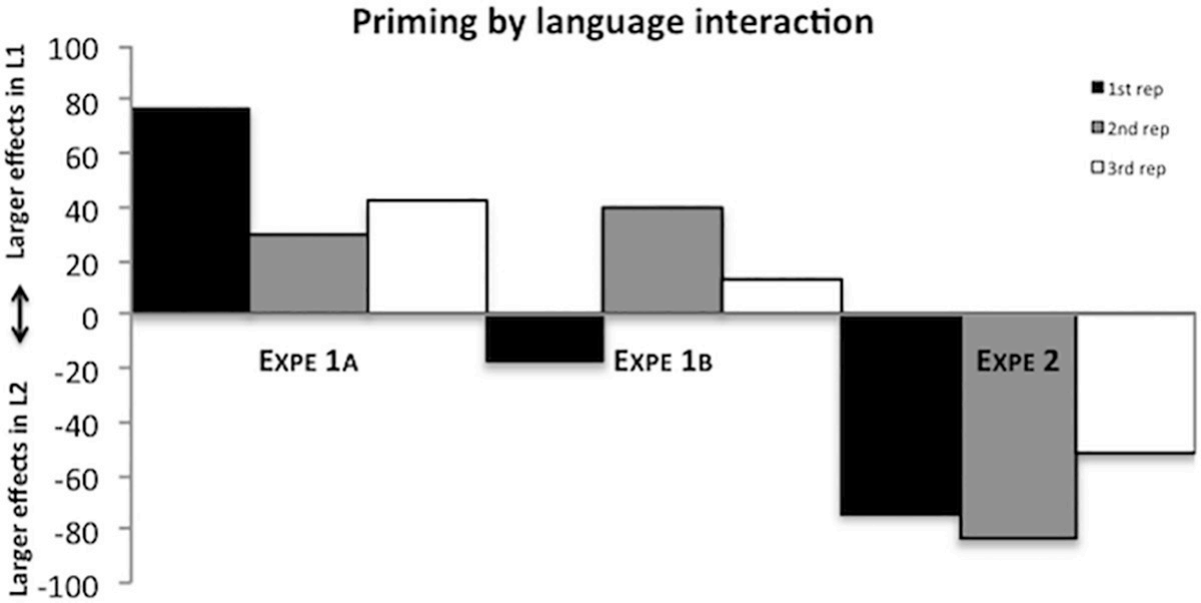
Difference in translation interference effects in ms between L1 and L2 naming (i.e., priming*language interaction) for non-cognates of Experiment 1a, 1b and 2. Positive numbers indicate a larger effect in L1 naming, negative numbers indicate a larger effect in L2 naming.

We observed that producing picture names in L3 was detrimental for subsequent naming of the translations in L2, but not significantly so for naming translations in L1. In combination with the results of Experiments 1a and 1b showing that learning is error-based (see Fig 2), these results directly support a competitive model of lexical selection: the strengthening of L3 words had a larger impact on subsequent L2 naming than on L1 naming because L2 words are already relatively weak and thus more vulnerable to competition compared to L1 words. On the contrary, these results are not compatible with the notion of competitor weakening (as the main mechanism behind translation interference). Under this view, the more strongly co-activated L1 words should have required more weakening than L2 words during the production in L3, resulting in larger translation interference during subsequent L1 naming compared to L2 naming. Do note, as mentioned in the Introduction, that our results are not necessarily incompatible with a model that implements both target strengthening and competitor weakening such as presented in the model of Oppenheim et al. [4]. That is, it remains perfectly plausible that lexico-semantic weight changes also rely on competitor weakening, as long as (long-lasting) semantic interference effects as observed in the present study rely mainly on target strengthening and thus are explained in terms of lexical competition. The latter is the main contribution of our study.

## General discussion

In this study, we investigated two fundamental properties of word production: First, we tested the hypothesis that the persistent lexico-semantic connection weight adjustments associated to the production of a word (i.e., learning) are sensitive to the difference in initial and desired activation of the word to be produced (i.e., error-based). Second, we tested the hypothesis that the selection of a word is sensitive to the activation levels of other related words (i.e., competitive lexical selection). We conducted three experiments in which participants first named pictures or words in one of their languages, and then named the same set of pictures (old set) and a new set in another language. The translation interference effect on RTs was measured, paying special attention to differences due to the degree of error in the different response languages in both parts (high vs. low named first vs. second).

Experiments 1a and 1b revealed that (a) regardless of the repetition or not of visual input across the experimental parts, naming non‐cognates in one language was detrimental for subsequent naming of translations in the other language, especially when the weaker language (L2) was used in the Learning phase and the stronger language (L1) in the Testing phase; and (b) naming cognates in one language facilitated production of translations in the other language. Experiment 2 showed that naming in a weak language (L3) was more detrimental for subsequent naming of translations in a second language (L2) than in the dominant and strongest language (L1). Taken together, these results suggest that produced words are persistently strengthened in proportion to the difference between their actual initial activation and the desired activation (i.e., error-based learning), and these strengthened words act as stronger competitors for a rather extended period of time when a related word has to be selected later on (i.e., lexical competition).

Note that our experimental rationale hinges on the assumptions that (a) translation equivalent pairs across languages are processed similarly to regular semantic competitors; and (b) proficiency and/or frequency of use effects of multilingual speakers’ different languages map directly onto a different degree of pre-selection error.

Concerning the assumption that translation equivalents are processed similarly to regular semantic competitors, one might wonder to what extent the processes required to restrict language production to a single language (i.e., bilingual language control) might interact with the effects of interest. Relevant to this issue, studies investigating semantic competitor effects across different languages suggest that effects of lexico-semantic competition can be reliably observed between languages [23, 57]. That is, should bilinguals require a specific mechanism of language control, such mechanism does not seem to preclude effects of cross-language lexico-semantic competition. Moreover, our findings may be used to constrain models of bilingual language control. The most extended view is that bilingual speakers inhibit words belonging to the unintended language [58–59], applying an amount of inhibition proportional to the degree of co-activation of the words from the unintended language. This tenet has received most of its support from studies observing asymmetrical switch costs in naming paradigms where speakers name pictures in both of their languages: naming in L1 entails a larger cost after L2 naming than vice versa, because L1 was more coactive during L2 naming than vice versa [60–61]. However, according to this logic, when speaking in an L3, the presumably strongly co-activated L1 words would have to be inhibited to a greater extent than the presumably weaklier co-activated L2 words [62]. If this were the case, we should have observed a larger cost in subsequent L1 naming compared to L2 naming in our Experiment 1 (in this sense, the notion of proportional language inhibition–being higher for strong representations–is conceptually similar to the notion of competitor weakening). Thus, our results suggest either that different mechanisms of language control are involved in contexts where both languages are used (as in language switching studies) and contexts where only one language is used (as in the present study), or that an alternative interpretation of this data pattern of asymmetrical switch costs is possible: because L2 words are weaker than L1 words, L2 words are relatively more strengthened during L2 speech than L1 words during L1 speech. This means that afterwards when switching languages, and in comparison to a baseline situation, L2 words will have gained more power as lexical competitors for L1 production than L1 words for L2 production. Note that this explanation entails that bilingual language control in essence could be reduced to the same type of mechanisms that all speakers use to prevent intrusions from undesired lexical competitors [23, 31–32, 39, 63–66].

Concerning the assumption of the possibility to map proficiency or frequency of use onto the degree of error present during lexical selection, several studies support the notion that while differences in proficiency and/or frequency of use across bilingual speakers languages are likely to impact the production process at several (or all) stages, the processing level at which these differences are likely to emerge is at the lexical level [30–32]. That, together with the fact that bilinguals name slower in their non-dominant than dominant language even when highly-proficient and having learned the L2 very early in life [29–32], supports the assumption that L1 has less error than L2 (and obviously less than the weak L3). Furthermore, the latter is additionally supported by the current data where in all experiments L1 produced the fastest naming latencies (even for the old items) (see Tables 2, 3 and 5). Clearly, if for some reason our participants would have reversed language dominance or particularities of the current design would induce such reversed dominance, then one would not have predicted that the naming latencies of L1 are the fastest across the board. In sum, both the previous literature and the current data confirm that mapping language proficiency/frequency of use onto degree of error is correct.

In what regards lexical competition, how might our study be reconciled with other empirical observations that seem to fit better with a model in which semantic interference arises as a consequence of competitor weakening [15, 24, 41]? First, it should be noted that some of this evidence might be inconsistent with lexical competition as implemented by a specific model, but not necessarily with the general notion of lexical competition [24]. Since this study is not committed to a particular account of lexical selection, such studies will not be discussed here. Concerning the few studies that report direct evidence against lexical competition, part of these differences might be accounted for through the different kind of stimuli used. For instance, Navarrete and colleagues [15, 41] found that semantic interference transferred from picture naming to word plus determiner naming but not vice versa [45]. Since both the tested naming modalities entailed lexical access, they argued that their findings are not consistent with a lexical locus of the semantic interference effect. Instead, along similar lines as Oppenheim and colleagues, they proposed that the semantic interference arises due to an incremental weakening in semantic-to-lexical connections that is exclusive to semantically mediated lexical access (e.g., picture naming but not word plus determiner naming). Here in our experiment 1b we did observe a transfer of competitor effects from word plus determiner naming onto picture naming, rendering the explanation given by Naverrete and colleagues unlikely (see also the Discussion-section of Experiments 1a and 1b, and [45]).

Finally, before concluding (and as correctly pointed out by a reviewer), our study relied on the error-based learning framework as specified by specific lexical selection models in the speech production literature (e.g., 2, 4–5) to generate the design and subsequent predictions. This does not necessarily mean that error-based learning is the only learning or attentional mechanism that can capture our data and does not preclude that other explanations related to ‘cognitive effort’ can explain our results. For example, assuming that during the learning phase top-down attention needs to be allocated more strongly for weaker representations (such as for L2 or L3 words) than strong ones (such as L1 words) [39, 67], it is conceivable that this “degree of attentional effort” has consequences for the amount of top-down processing on the subsequent Test phase; a dynamic which may predict similar results. That said, two considerations are important: First, the notion of ‘cognitive or attentional effort’ need not be in contradiction with the notion of ‘error-based learning’. Error-based learning can perfectly be conceptualized as one specific mechanistic implementation of how ‘cognitive effort’ can affect (language) processing after learning. Second, focusing in the current study on the error-based learning framework seems fair. This is because reliance on this framework in other studies of language production have been essential to explain how lexical selection and semantic interference do not require an explanation in terms of lexical competition. “Playing the game by the same rules” we here observe that reliance on that very same framework does require a lexical system which is competitive. We believe this is an important contribution, not only to constrain those speech production models that specifically implement error-based learning, but for the long-standing debate regarding lexical competition in the field in general.

## Conclusion

The results of the three experiments reported here provide evidence for a dynamics of language production–whether bilingual or monolingual–in which words selected for production are persistently strengthened in an error-based fashion, rendering them stronger competitors when semantically related words have to be produced later on. In this manner, and contrary to current claims in the field, our data supports the notion that lexical selection in word production is a competitive process.

## Supporting information

S1 Table Target words used in Experiments 1a, 1b and 2. (DOCX)

S1 File Information concerning the language history of participants. (DOCX)

S2 File Means and statistical analyses of error-rates in Experiment 1a, 1b and 2. (DOCX)

S3 File Response time and accuracy data of Experiment 1a for the 30 participants who named in Catalan in part A and Spanish in part B. (XLSX)

S4 File Response time and accuracy data of Experiment 1a for the 29 participants who named in Spanish in part A and Catalan in part B. (XLSX)

S5 File Response time and accuracy data of Experiment 1b for the 30 participants who named in Catalan in part A and Spanish in part B. (XLSX)

S6 File Response time and accuracy data of Experiment 1b for the 30 participants who named in Spanish in part A and Catalan in part B. (XLSX)

S7 File Response time and accuracy data of Experiment 2 for the 27 participants who named in English in part A and Spanish in part B. (XLSX)

S8 File Response time and accuracy data of Experiment 2 for the 27 participants who named in English in part A and Catalan in part B. (XLSX)
