# Supplementary material for: Error-based learning and lexical competition in word production: Evidence from multilingual naming
Source: PLoS One. 2019 Mar 22;14(3):e0213765. doi: 10.1371/journal.pone.0213765 (PMC6430390; doi:10.1371/journal.pone.0213765)
Supplement: S1 Table — (DOCX) [file pone.0213765.s001.docx]

Appendix B. Target words uses in Experiments 1a, 1b and 2

Target words used for Experiments 1a and 1b

| NON-COGNATES | | COGNATES | | NON-COGNATES | | COGNATES | |
| --- | --- | --- | --- | --- | --- | --- | --- |
| *Catalan* | *Spanish* | *Catalan* | *Spanish* | *Catalan* | *Spanish* | *Catalan* | *Spanish* |
| mussol | búho | girafa | jirafa | porc | cerdo | orella | oreja |
| maduixa | fresa | ovella | oveja | dit | dedo | ós | oso |
| papallona | mariposa | patí | patín | taronja | naranja | arbre | árbol |
| guineu | zorro | plàtan | plátano | ull | ojo | casc | casco |
| Mitjó | calcetín | ratolí | ratón | branca | rama | faldilla | falda |
| destral | hacha | escombra | escoba | llit | cama | rellotge | reloj |
| baldufa | peonza | guant | guante | clau | llave | telèfon | teléfono |
| forquilla | tenedor | tanc | tanque | mocador | pañuelo | vestit | vestido |
| esquirol | ardilla | cocodril | cocodrilo | fulla | hoja | cavall | caballo |
| Cuc | gusano | drac | dragón | ocell | pájaro | nas | nariz |
| enciam | lechuga | elefant | elefante | ganivet | cuchillo | avió | avión |
| préssec | melocotón | pingüí | pingüino | ulleres | gafas | camió | camión |
| Ànec | pato | cullera | cuchara | taula | mesa | globus | globo |
| pastanaga | zanahoria | llapis | lápiz | formatge | queso | pa | pan |
| galleda | cubo | pom | pomo | xarxa | red | pilota | pelota |
| guardiola | hucha | violí | violín | espelma | vela | plat | plato |
| sargantana | lagartija | camell | camello | ou | huevo | braç | brazo |
| queixal | muela | formiga | hormiga | pluja | lluvia | flor | flor |
| granota | rana | tigre | tigre | poma | manzana | gat | gato |
| Raïm | uva | bombeta | bombilla | gos | perro | núvol | nube |
| raspall | cepillo | escriptori | escritorio | butxaca | bolsillo | anell | anillo |
| Aixeta | grifo | martell | martillo | cadira | silla | banc | banco |
| Paella | sartén | pinzell | pincel | barret | sombrero | caixa | caja |
| Xiulet | silbato | trineu | trineo | finestra | ventana | cinturó | cinturón |
|  |  |  |  |  |  |  |  |

Target words used for Experiment 2

|  | | |  | | |
| --- | --- | --- | --- | --- | --- |
| *English* | *Catalan* | *Spanish* | *English* | *Catalan* | *Spanish* |
| Frog | granota | rana | chair | cadira | silla |
| strawberry | maduixa | fresa | finger | dit | dedo |
| butterfly | papallona | mariposa | orange | taronja | naranja |
| Fox | guineu | zorro | eye | ull | ojo |
| Sheep | ovella | oveja | ear | orella | oreja |
| Rabbit | conill | conejo | box | caixa | caja |
| banana | plàtan | plátano | airplane | avió | avión |
| mouse | ratolí | ratón | clock | rellotge | reloj |
| Pillow | coixí | almohada | bed | llit | cama |
| Bat | rat-penat | murciélago | key | clau | llave |
| Duck | ànec | pato | cheese | formatge | queso |
| Carrot | pastanaga | zanahoria | egg | ou | huevo |
| Glove | guant | guante | dress | vestit | vestido |
| Spoon | cullera | cuchara | ring | anell | anillo |
| Pencil | llapis | lápiz | tree | arbre | árbol |
| Spider | aranya | araña | bread | pa | pan |
| lighter | encenedor | mechero | apple | poma | manzana |
| Owl | mussol | búho | dog | gos | perro |
| Grape | raïm | uva | pig | porc | cerdo |
| Kite | estel | cometa | window | finestra | ventana |
| Desk | escriptori | escritorio | ball | pilota | pelota |
| Clown | pallasso | payaso | arm | braç | brazo |
| Beetle | escarabat | escarabajo | horse | cavall | caballo |
| Cake | pastís | pastel | bear | ós | oso |
