# Supplementary material for: Error-based learning and lexical competition in word production: Evidence from multilingual naming
Source: PLoS One. 2019 Mar 22;14(3):e0213765. doi: 10.1371/journal.pone.0213765 (PMC6430390; doi:10.1371/journal.pone.0213765)
Supplement: S1 File — (DOCX) [file pone.0213765.s002.docx]

Appendix A. Information concerning the language history of participants

Participants were recruited based on their language dominance: they could only participate if they considered themselves as dominant in L1, spoke in L1 with the mother since birth, and considered their use of L1 to be more frequent than that of L2 (for the accuracy of such subjective measures to determine language dominance see Gollan, Weissberger, Runnqvist, Montoya & Cera, 2012). The age and self-assessed proficiency scores of the participants of all the experiments as well as their estimated daily use of Spanish and Catalan and estimated onset of use of each of their languages are presented in Table 1. The mean age is given in years. All scores were obtained through a questionnaire filled out by the participants. The proficiency scores are on a 4-point scale, where 4 = native-speaker level; 3 = advanced level; 2 = medium level; and 1 = low level of proficiency. The self-assessment index represents the average of the participants’ responses in four domains (speech comprehension, speech production, reading, and writing). The percentage of daily use represents the average of the estimated use when growing up and estimated use currently. The onset of Spanish and Catalan represents the average between the onset of listening and speaking while the onset of English represents the age at which participants reported that they started learning English.

|  | Age | Span. prof. | Cat. prof. | Eng. prof. | %use Span. | %use Cat. | Onset Span. | Onset Cat. | Onset Eng. |
| --- | --- | --- | --- | --- | --- | --- | --- | --- | --- |
| Expe1 | 22.2 (3.4) | 3.99 (0.06) | 3.81 (0.31) | 2.24 (0.74) | 71 (9) | 29 (9) | 0.8^a^ (0.9) | 3.4^b^ (2.1) | 7.6^c^ (2.1) |
| Expe2a^d^ | 21.1 (3.6) | 4.00 (0.00) | 3.87 (0.26) | 2.80 (0.69) | 64 (11) | 36 (11) | 0.9 (0.7) | 2.9 (1.7) | 6.9^e^ (2.1) |
| Expe2b | 20.6 (3.2) | 3.97 (0.15) | 3.83 (0.23) | 2.54 (0.69) | 68 (15) | 32 (15) | 0.6 (0.7) | 2.6 (1.6) | 6.7^f^ (2.5) |

(a) One participant could not provide an estimation of the onset of L1

(b) One participant could not provide an estimation of the onset of L2

(c) One participant answered that he/she started learning English during his/her primary education. One participant did not answer this question.

(d) The averages of Experiment 1 stem from 19 questionnaires; 38 questionnaires were lost. Nevertheless, students could only participate in the experiment if they considered themselves to be dominant in Spanish, estimated that they used Spanish more frequently than Catalan and spoke in Spanish with their mother or both their parents. Before the experimental session their eligibility regarding these criteria was assessed.

(e) Five participants answered that they started learning English during their primary education, but could not provide an exact age. One participant did not answer this question.

(f) Four participants answered that they started learning English during their primary education, two during their secondary education and two during their period in school, but could not provide an exact age. Five participants did not answer this question.
