# Supplementary material for: Error-based learning and lexical competition in word production: Evidence from multilingual naming
Source: PLoS One. 2019 Mar 22;14(3):e0213765. doi: 10.1371/journal.pone.0213765 (PMC6430390; doi:10.1371/journal.pone.0213765)
Supplement: S2 File — (DOCX) [file pone.0213765.s003.docx]

Means and statistical analyses of error-rates in Experiment 1a and 1b

**Part A.**

Table 3a. Error-rates for Part A in Experiment 1a and 1b. Numbers in parenthesis represent the mean standard error.

|  | **L1 1^st^ rep.** | **L1 2^nd^ rep.** | **L1 3^rd^ rep.** | **L2 1^st^ rep.** | **L2 2^nd^ rep.** | **L2 3^rd^ rep.** |
| --- | --- | --- | --- | --- | --- | --- |
| P non-cog | 15,0 (1,3) | 6,9 (1,2) | 4,7 (1,0) | 25,3 (1,3) | 10,9 (1,1) | 8,1 (1,0) |
| P cog | 10,0 (0,9) | 3,6 (0,7) | 3,0 (0,6) | 12,0 (0,9) | 4,6 (0,7) | 3,5 (0,6) |
| W non-cog | 1,6 (1,3) | 1,0 (1,1) | 1,3 (1,0) | 3,6 (1,3) | 1,6 (1,1) | 1,2 (1,0) |
| W cog | 1,0 (0,9) | 0,8 (0,7) | 0,7 (0,6) | 2,3 (0,9) | 1,4 (0,7) | 0,9 (0,6) |

Repetition, Cognate status and Language interacted significantly by subjects (F1(2,228)=5.826, MSE=.1, p=.006; F2(2,752)=1.899, MSE=.6, p=.166). The Cognate status*Language interaction was only significant in the first repetition by subjects (F1(2,228)=14.614, MSE=.2, p<.001; F2(1,380)=1.977, MSE=2.4, p=.161). In the second repetition there was a main effect of Cognate status (F1(1,114)=26.170, MSE=.1, p<.001; F2(1,380)=11.010, MSE=.6, p=.001) and a marginal effect of Language (F1(1,114)=3.685, MSE=.4, p=.057; F2(1,380)=3.679, MSE=.6, p=.056). In the third repetition there was a main effect of Cognate status (F1(1,114)=17.776, MSE=.1, p<.001; F2(1,380)=9.006, MSE=.3, p=.003). There were also interactions between Repetition, Cognate status and Experiment (F1(2,228)=13.116, MSE=.1, p<.001; F2(2,752)=5.049, MSE=.6, p=.018), and Cognate status, Experiment and Language by subjects (F1(2,228)=7.929, MSE=.1, p=.006; F2(1,376)=1.947, MSE=1.9, p=.164). In picture naming there was a Repetition*Cognate status interaction (F1(2,112)=18.969, MSE=.2, p<.001; F2(2,376)=7.021, MSE=1.2, p=.006), and a Cognate status*Language interaction by subjects (F1(1,56)=9.414, MSE=.5, p=.003; F2(1,188)=2.256, MSE=3.5, p=.135). In word naming these interactions were not significant, but there was a main effect of Repetition (F1(2,116)=9.229, MSE=.0, p<.001; F2(2,376)=7.769, MSE=.1, p=.001), and by subjects there were main effects of Cognate status (F1(1,58)=3.843, MSE=.1, p=.055; F2(1,188)=1.500, MSE=.3, p=.222) and Language (F1(1,58)=6.424, MSE=.1, p=.014; F2(1,188)=2.105, MSE=.3, p=.149).

**Part B.**

Table 3b. Error-rates for Part B in Experiment 1a. Numbers in parenthesis represent the mean standard error.

| **Non-cog** | **L1 1^st^ rep.** | **L1 2^nd^ rep.** | **L1 3^rd^ rep.** | **L2 1^st^ rep.** | **L2 2^nd^ rep.** | **L2 3^rd^ rep.** |
| --- | --- | --- | --- | --- | --- | --- |
| NEW items | 14,0 (2,2) | 4,1 (1,5) | 4,1 (1,5) | 24,8 (2,3) | 8,4 (1,5) | 5,9 (1,5) |
| OLD items | 10,2 (1,9) | 5,0 (1,9) | 4,7 (1,6) | 19,0 (1,9) | 9,8 (1,9) | 8,3 (1,7) |

| **Cog** | **L1 1^st^ rep.** | **L1 2^nd^ rep.** | **L1 3^rd^ rep.** | **L2 1^st^ rep.** | **L2 2^nd^ rep.** | **L2 3^rd^ rep.** |
| --- | --- | --- | --- | --- | --- | --- |
| NEW items | 7,6 (1,5) | 3,2 (1,2) | 2,6 (1,0) | 11,1 (1,5) | 5,4 (1,2) | 3,7 (1,0) |
| OLD items | 4,0 (1,2) | 2,5 (1,1) | 2,5 (1,0) | 6,5 (1,2) | 3,3 (1,1) | 3,3 (1,0) |

Table 3c. Error-rates for Part B in Experiment 1b. Numbers in parenthesis represent the mean standard error.

| **Non-cog** | | **L1 1^st^ rep.** | | **L1 2^nd^ rep.** | | **L1 3^rd^ rep.** | | **L2 1^st^ rep.** | | **L2 2^nd^ rep.** | | **L2 3^rd^ rep.** | |
| --- | --- | --- | --- | --- | --- | --- | --- | --- | --- | --- | --- | --- | --- |
| NEW items | | 17,1 (2,2) | | 12,1 (1,5) | | 10,3 (1,5) | | 21,9 (2,2) | | 13,2 (1,5) | | 12,7 (1,5) | |
| OLD items | | 17,5 (1,9) | | 11,3 (1,9) | | 8,6 (1,6) | | 20,3 (1,9) | | 14,1 (1,9) | | 11,4 (1,6) | |
| **Cog** | | **L1 1^st^ rep.** | | **L1 2^nd^ rep.** | | **L1 3^rd^ rep.** | | **L2 1^st^ rep.** | | **L2 2^nd^ rep.** | | **L2 3^rd^ rep.** | |
| NEW items | | 11,6 (1,5) | | 4,6 (1,2) | | 3,8 (1,0) | | 11,0 (1,5) | | 6,8 (1,2) | | 5,7 (1,0) | |
| OLD items | | 7,4 (1,2) | | 5,2 (1,1) | | 4,2 (1,0) | | 9,3 (1,2) | | 5,1 (1,1) | | 3,6 (1,0) | |

There was an interaction between Repetition, Priming, Cognate status and Language (F1 (2, 230)=3.450, MSE=.003, p=.039; F2 (2, 712)=2.803, MSE=.006, p=.074), between Repetition, Cognate status and Experiment (F1 (2, 230)=7.120, MSE=.003, p=.002; F2 (2, 712)=3.169, MSE=.014, p=.066), and between Repetition, Priming and Experiment (F1 (2, 230)=5.394, MSE=.004, p=.011; F2 (2, 712)=5.527, MSE=.006, p=.008). As a next step we analyzed each repetition separately. In the first repetition there were significant main effects of Priming (F1 (1, 115)=12.653, MSE=.009, p=.001; F2 (1, 356)=16.092, MSE=.011, p>.001) and Experiment (F1 (1, 115)=4.407, MSE=.015, p=.038; F2 (1, 356)=1.572, MSE=.060; p=.211), and an interaction between Cognate status and Language (F1 (1, 115)=11.745, MSE=.006, p=.001; F2 (1, 356)=1.775, MSE=.060, p=.184). In the second repetition there was a main effect of Language (F1 (1, 115)=5.214, MSE=.011, p=.024; F2 (1, 356)=3.867, MSE=.024, p=.050), and an interaction between Cognate status and Experiment (F1 (1, 115)=10.480, MSE=.005, p=.002; F2 (1, 356)=2.917, MSE=.024, p=.089). In the third repetition there was an interaction between Cognate status and Experiment (F1 (1, 115)=12.548, MSE=.003, p=.001; F2 (1, 356)=3.003, MSE=.020, p=.084).

Means and statistical analyses of error-rates in Experiment 2

**Part A.**

Table 4a. Error-rates for Part A in Experiment 2. Numbers in parenthesis represent the mean standard error.

|  | **L3 1^st^ rep.** | **L3 2^nd^ rep.** | **L3 3^rd^ rep.** |
| --- | --- | --- | --- |
|  | 12,2 (1,6) | 10,7 (1,6) | 9,1 (1,5) |

There was a main effect of repetition (F1(2,104)=18.851, MSE=7.074, p<.001; F2(2,188)=13.420, MSE=19.243, p<.001).

**Part B.**

Table 4b. Error-rates for Part B in Experiment 2. Numbers in parenthesis represent the mean standard error.

|  | **L1 1^st^ rep.** | **L1 2^nd^ rep.** | **L1 3^rd^ rep.** | **L2 1^st^ rep.** | **L2 2^nd^ rep.** | **L2 3^rd^ rep.** |
| --- | --- | --- | --- | --- | --- | --- |
| NEW items | 10,0 (1,6) | 7,1 (1,4) | 6,1 (1,3) | 12,6 (1,6) | 9,0 (1,4) | 6,8 (1,3) |
| OLD items | 7,1 (1,9) | 3,9 (1,1) | 4,9 (1,3) | 11,0 (1,9) | 5,1 (1,1) | 5,8 (1,3) |

There were main effects of Priming (F1(1, 52) =3.917, MSE=.011, p=.053, F2 (1, 90)=3.293, MSE=.018, p=.073) and Repetition (F1(2, 104) =25.113, MSE=.003, p<.001, F2 (2, 180)=23.589, MSE=.005, p<.001).
